# Rectally administered indomethacin to prevent post-ESWL-pancreatitis (RIPEP): study protocol for a randomized controlled trial

**DOI:** 10.1186/s13063-017-2250-7

**Published:** 2017-11-02

**Authors:** Yang-Yang Qian, Hui Chen, Xin-Ying Tang, Xi Jiang, Wei Qian, Wen-Bin Zou, Lei Xin, Bo Li, Yan-Fen Qi, Liang-Hao Hu, Duo-Wu Zou, Zhen-Dong Jin, Dong Wang, Yi-Qi Du, Luo-Wei Wang, Feng Liu, Zhao-Shen Li, Zhuan Liao

**Affiliations:** 1 Department of Gastroenterology, Digestive Endoscopy Center, Changhai Hospital, the Second Military Medical University, 168 Changhai Road, Shanghai, 200433 China; 2 Digestive Endoscopy Center, Changhai Hospital, the Second Military Medical University, Shanghai, China; 3 Department of Anesthesiology, Changhai Hospital, the Second Military Medical University, Shanghai, China; 4 Shanghai Institute of Pancreatic Diseases, Shanghai, China; 5 National Clinical Research Center of Digestive Diseases, Shanghai, China

**Keywords:** ESWL, Prophylaxis, Trial, Indomethacin, Post-ESWL pancreatitis

## Abstract

**Background:**

Pancreatic extracorporeal shock wave lithotripsy (P-ESWL) is the first-line therapy for large pancreatic duct stones. Although it is a highly effective and safe procedure for the fragmentation of pancreatic stones, it is still not complication-free. Just like endoscopic retrograde cholangiopancreatography (ERCP), pancreatitis is the most common complication. To date, nonsteroidal anti-inflammatory drugs (NSAIDs) have proven to be the only effective prophylactic medication for post-ERCP pancreatitis and the European, American and Japanese Society for Gastrointestinal Endoscopy guidelines have recommended prophylactic rectally administered indomethacin for all patients undergoing ERCP. Given the little research about effective prevention for post P-ESWL pancreatitis, we aim to determine whether rectally administered indomethacin can reduce post-ESWL-pancreatitis.

**Methods/design:**

The RIPEP study is a prospective, randomized, double-blinded, placebo-controlled trial. One thousand three hundred and seventy patients with chronic pancreatitis and pancreatic stones (>5 mm in diameter) treated with P-ESWL at Changhai Hospital will be randomly allocated to rectally administered indomethacin or placebo therapy before the procedure. The primary endpoint is the incidence of post-ESWL pancreatitis. Secondary endpoints include the severity of pancreatitis, occurrence rate of asymptomatic hyperamylasemia and other complications.

**Discussion:**

The RIPEP trial is designed to show that rectally administered indomethacin reduces the development and severity of post-ESWL pancreatitis and benefits patients treated with P-ESWL.

**Trial registration:**

ClinicalTrials.gov, ID: NCT02797067. Registered on 17 November 2016.

**Electronic supplementary material:**

The online version of this article (doi:10.1186/s13063-017-2250-7) contains supplementary material, which is available to authorized users.

## Background

Chronic pancreatitis encompasses a wide range of progressive fibro-inflammatory processes of the exocrine pancreas that eventually lead to damage of the gland, leading to abdominal pain, endocrine (diabetes) and exocrine insufficiency (steatorrhea). Pancreatic duct stones, a common complication of chronic pancreatitis, develop during the natural course of disease and are observed in 90% patients [1]. Current treatment options include endoscopic therapy, extracorporeal shock wave lithotripsy (ESWL) and surgery. ESWL disintegrates the stones as a compensatory role thus facilitating main pancreatic duct (MPD) sphincterotomy, stricture dilatation, stone extraction and MPD stenting during endoscopic retrograde cholangiopancreatography (ERCP) [2, 3]. The European Society of Gastrointestinal Endoscopy (ESGE) recommends ESWL as a first step, immediately followed by endoscopic extraction of stone fragments for treating patients with uncomplicated, painful chronic pancreatitis and radiopaque stones of 5 mm or more obstructing the MPD [4]. Although proved as safe, effective and noninvasive in stone fragmentation [5–8], ESWL can still cause adverse events, which can be classified as complications and transient adverse events (TAEs), depending on the severity. Based on previous published studies, major complications were classified into five groups: post-ESWL pancreatitis, bleeding, infection, steinstrasse and perforation [6, 9]. It is reported that the rate of post-ESWL pancreatitis ranges from 6.3 to 12.5% [1]. According to previous data in our center, post-ESWL pancreatitis was the most common complication, with an overall occurrence rate of 6.8% for the first P-ESWL sessions. Some cases may need specific medical treatment or prolonged hospitalization, high medical expenditure and may even be life-threatening [9].

Compared to ESWL, complications of ERCP have been widely studied and the prevention strategies have been particularly analyzed by prior studies. Post-ERCP pancreatitis (PEP) also proved to be the most common complication with a reported incidence ranging from 3.6 to 15.1% in large-scale studies [10]. To date, various prophylactic procedures have been applied while prophylactic pancreatic stent (PPS) placement and rectally administered nonsteroidal anti-inflammatory drugs (NSAIDs) are promising for decreasing the rate and severity of PEP [11–16]. The strongly prophylactic effect of NSAIDs has prompted the European Society of Comparative Gastroenterology (ESGE), the Japanese Society of Hepato-Biliary-Pancreatic Surgery and The Standards of Practice Committee of the American Society for Gastrointestinal Endoscopy (ASGE) to recommend the intrarectal administration of NSAIDs in all cases undergoing ERCP without contraindications [17–19]. Furthermore, Luo et al. have conducted a multicenter RCT and demonstrated that pre-procedural rectal administration of indomethacin in unselected patients reduced the overall occurrence of PEP compared with a risk-stratified and post-procedural strategy [20].

As there are few researches regarding the incidence and prophylaxis of post-ESWL complications and given the potential clinical and economic benefit, we designed this prospective, randomized, double-blinded, placebo-controlled trial to investigate whether rectally administered indomethacin can effectively decrease the incidence and severity of post-ESWL pancreatitis as well as any associated adverse events, thus benefiting patients treated with P-ESWL.

## Methods/design

### Design

The RIPEP trial is a prospective, randomized, double-blinded, placebo-controlled trial designed to show whether rectally administered indomethacin reduces the incidence of post-ESWL pancreatitis. Patients treated with P-ESWL at Changhai Hospital from May 2016 have been being randomly allocated to premedication with rectally administered indomethacin or glycerin, within 30 min before the procedure after randomization. For patients undergoing more than one P-ESWL session, only the first procedure will be included in the study. Ethical approval has been obtained from Changhai Institutional Review Board. The Standard Protocol Items: Recommendations for Interventional Trial (SPIRIT) Checklist is provided as Additional file 1.

### Study population

**Fig. 1:**
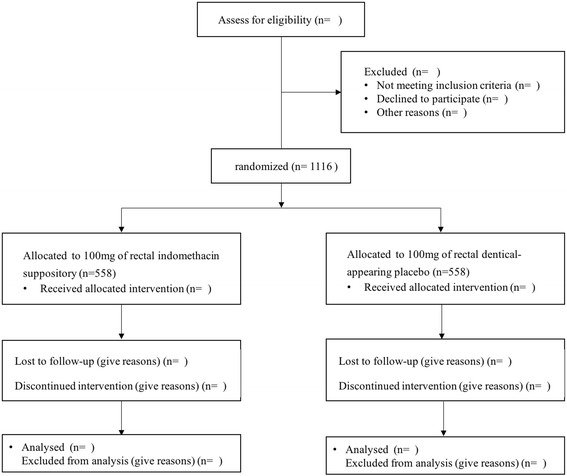
Study flowchart

This prospective study will be performed at Changhai Hospital. All adult patients admitted with chronic pancreatitis will be assessed for eligibility during their hospital admission. If patients are classified as having pancreatic stones (5 mm or more in diameter) and fulfill all inclusion and exclusion criteria, they will be randomized (with a 1:1 ratio) to the rectal administration of indomethacin group or the placebo group (Fig. 1).

The eligibility criteria are listed in Table 1. All patients (aged 18 years or older) with chronic pancreatitis treated with P-ESWL for pancreatic stones at Changhai Hospital will be eligible for enrollment. Study group personnel will explain the study to the patients and written informed consent will be obtained from each patient. Each participant has the right to refuse or withdraw from the study without giving any reasons. ESWL will be performed with at least one large pancreatic stone (5 mm or more in diameter).

**Table 1:** Eligibility criteria

Any patient with chronic pancreatitis and pancreatic stones (5 mm or more in diameter) undergoing P-ESWL who is at least 18 years old, provides informed consent, and:
Exclusion criteria
Contraindications to ESWL
Suspected or established malignancy
Pancreatic ascites
Receiving NSAIDs within 7 days
Contraindication to NSAIDs (including gastrointestinal hemorrhage within 4 weeks or renal dysfunction with serum creatinine > 120 μmol/L)
Presence of coagulopathy or received anticoagulation therapy within 3 days
Acute pancreatitis within 3 days
Known active cardiovascular or cerebrovascular disease
Unwilling or unable to provide consent
Pregnant or breastfeeding women
Being without a rectum (i.e., status post-total proctocolectomy)

*ESWL* extracorporeal shock wave lithotripsy, *NSAIDs* nonsteroidal anti-inflammatory drugs, *P-ESWL* pancreatic extracorporeal shock wave lithotripsy

Patients will be excluded if they have contraindications to ESWL, have contraindication to NSAIDs (including gastrointestinal hemorrhage within 4 weeks or renal dysfunction with serum creatinine > 120 μmol/L), have coagulopathy or receive anticoagulation therapy within 3 days or NSAIDs within 7 days, have acute pancreatitis within 3 days, have known active cardiovascular or cerebrovascular disease, and those who are suspected or established with malignancy, pancreatic ascites, allergic to NSAIDs, unwilling or unable to provide consent, and pregnant or breastfeeding women, or those without a rectum (i.e., status post-total proctocolectomy). Contraindications for ESWL will be determined by endoscopists or anesthesiologists before P-ESWL.

### Treatment protocol

Before the procedure, if all inclusion criteria have been met and none of the exclusion criteria are present, the subject will be randomized to receive either a 100-mg indomethacin suppository or identical-appearing placebo. The suppository will be administered within 30 min before the procedure by a trained research nurse blind to the type of suppository labeled with allocation sequence. Randomization will occur in a 1:1 fashion with a random number table generated by a statistician, making it possible that all the patients and staff endoscopists are all blinded to the treatment assigned to each participant.

P-ESWL will be performed by two gastroenterologists (HLH and CH) using an electromagnetic lithotripter with bi-dimensional fluoroscopic targeting facility. The pretreatment procedure is similar to that for ERCP. Intravenously administered remifentanil combined with dexmedetomidine is administered for analgesia during the procedure.

Patients are to be placed in the supine position or were tilted to their right side at an angle of 30°. The exposure is limited to a maximum of 5000 shock waves per session. An intensity ranging from 1 to 6 was used with a frequency of 60–120 shocks per min during the procedure. The duration of each session is 60–90 min. The fragmentation of the stones is monitored by fluoroscopy during the P-ESWL session.

### Data collection and follow-up

**Fig. 2:**
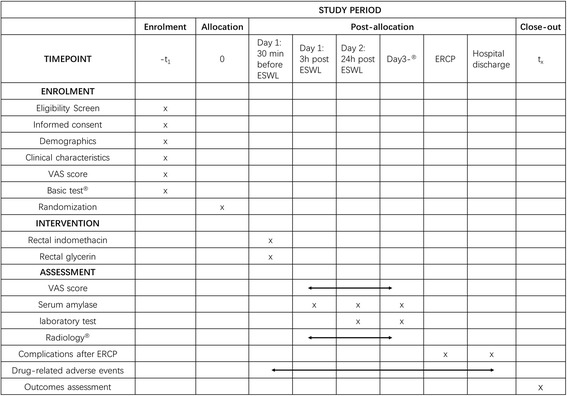
Schedule of enrollment, interventions and assessments. Basic test: laboratory tests including routine blood, urine and stool analysis, coagulation function, D-dimer, liver and kidney function, serum amylase, glycosylated hemoglobin (HbA1c),autoimmune-related indexes; electrocardiogram (ECG); radiology including chest X-ray, computed tomography (CT) and magnetic resonance imaging (MRI). Day 3: if extracorporeal shock wave lithotripsy (ESWL)-related complications occurred, patients will be follow-up after ESWL. Radiology: if new abdominal pain appears at any moment during the surveillance period, radiology may be carried out to confirm complications

After the P-ESWL, patients will be kept under surveillance for up to 24 h. A Visual Analogue Scale (VAS) before and after the procedure will be recorded. Serum amylase, routine hematology and biochemical tests will be measured in all study patients 3 and 24 h after the procedure and subsequently at clinical discretion. If new abdominal pain appears at any moment during the surveillance period, the amylase level will be measured and confirmed with imaging results. Detailed information can be found in Fig. 2. The goal of follow-up is to ascertain data necessary to adjudicate the outcomes. Clinical data with regard to baseline characteristics and outcomes will be collected during hospital admission using Case Report Forms (CRFs). Drug-related adverse events and management will also be collected and reported in the CRFs. CRFs will be filled out by study group personnel. Every 6 months, these data will be monitored by Changhai Institutional Review Board as the independent Data Monitoring Committee (IDMC) which is independent of the trial organizers.

### Outcomes

The primary outcome is whether 100 mg of rectally administered indomethacin compared with placebo would decrease the incidence of post-ESWL pancreatitis in all patients undergoing ESWL. The diagnosis of post-ESWL pancreatitis is established on consensus criteria, which was defined as new upper abdominal pain, an elevation in pancreatic enzymes to at least three times the normal level 24 h after the procedure, and requires admission or extension of planned admission for least 2 days. The secondary outcome was to identify the frequency (incidence) of moderate to severe post-ESWL pancreatitis, asymptomatic hyperamylasemia and other post-ESWL complications (including bleeding, infection, steinstrasse and perforation). Post-ESWL complications are also stratified as mild, moderate and severe depending mainly on the length of hospitalization and the need for invasive treatment (Table 2). Asymptomatic hyperamylasemia was defined as an increase in serum amylase compared with pre-ESWL levels and beyond the upper limit of the normal range but showing no related symptoms. The definition and stratification of other complications can be found in Table 2.

**Table 2:** Definitions of major complications of pancreatic extracorporeal shock wave lithotripsy

Complications	Mild	Moderate	Severe
Post-ESWL pancreatitis	Clinical pancreatitis, amylase at least three times the normal level at > 24 h after procedure, requires admission or extension of planned admission from 2 days to 3 days	Requires hospitalization of 4– 10 days	Hospitalization for > 10 days, pseudocyst formation, or intervention (percutaneous drainage or surgery)
Bleeding	Clinical evidence of bleeding, hemoglobin fall < 3 g, no transfusion	Transfusion of ≤ 4 units, no angiographic intervention, or surgery	Transfusion of ≥ 5 units, or intervention (angiographic or surgical)
Infection	>38 °C for 24–48 h	Requires > 3 days of hospital treatment	Abscess, septic shock, or intervention (percutaneous drainage or surgery)
Steinstrasse	Severe abdominal pain without other post-ESWL complications	Combined with other complications, or requires > 3 days of hospital treatment	Combined with other complications; hospitalization for > 10 days, or surgery
Perforation	Possible, or very slight leak of fluid, treatable with fluids and suction for ≤ 3 days	Any definite perforation treated medically for 4–10 days	Medical treatment for > 10 days, or intervention (percutaneous or surgical)

*ESWL* extracorporeal shock wave lithotripsy

### Statistical considerations

#### Sample size

The sample size calculation is based on our previous research on the incidence rate of post-ESWL pancreatitis. Post-ESWL pancreatitis occurs in 6.8% of the patients for the first P-ESWL sessions and accounting for 69.4% of complications [9]. The RIPEP trial is a superiority trial in which the sample size is based on the assumption that rectally administered indomethacin reduces the incidence of the primary endpoint by a relative reduction of 50%. This amounts to an expected incidence of post-ESWL in the rectally administered indomethacin group of 3.4%. Assuming a one-sided alpha of 0.025 and a power of 80%, 1370 patients (685 per arms) would be necessary to detect a 6.8 to 3.4% reduction of post-ESWL pancreatitis, including a possible withdrawal rate of 5%.

#### Descriptive statistics

For dichotomous data, frequencies will be presented. Continuous data will be presented as mean and standard deviation or median and interquartile range. The following patient characteristics before randomization will be described in the CRF involving age, sex, etiology, smoking status, abdominal pain pattern, complications, previous pancreatic surgery, previous ERCP, and clinical stage as listed in Table 3. The M-ANNHEIM clinical staging system is adapted from a previous classification of chronic pancreatitis which classifies patients according to pain pattern, pancreatic exocrine and endocrine insufficiency and the presence of severe complications (Table 4) [21]. Results will be reported according to the Consolidated Standards of Reporting Trials (CONSORT) Statement.

**Table 3:** Demographic and clinical characteristics of chronic pancreatitis (CP) patients in the study

Age	
At onset of chronic pancreatitis	
At diagnosis of chronic pancreatitis	
At presentation with pancreatic stone	
At first P-ESWL session	
Sex	
Smoking status	
Etiology	
Alcoholic CP	
Idiopathic CP	
Hereditary (familial) CP	
Metabolic	
Traumatic	
Others	
Pain pattern	
Painless	
Recurrent acute pancreatitis	
Recurrent abdominal pain (RP) without significant increase in serum amylase	
Recurrent acute pancreatitis or abdominal pain (RAP/P) without significant increase in serum amylase	
Chronic pancreatic pain (CPP)	
Complications	
Diabetes	
Steatorrhea	
Pseudocyst	
Ductus choledochus obstruction or stricture	
Duodenal stenosis	
Pancreatic fistula	
Portal hypertension	
Treatment history	
Previous ESWL	
Precious ERCP	
Successful drainage with previous ERCP	
Previous EPT	
Previous pancreatic duct stent implantation	
Pancreatic surgery history	
M-ANNHEIM clinical stages	
I a/b/c	
II a/b/c	
III a/b	
IV a/b	

*EPT ERCP* endoscopic retrograde cholangiopancreatography, *ESWL* extracorporeal shock wave lithotripsy

**Table 4:** M-ANNHEIM clinical staging of chronic pancreatitis

Asymptomatic chronic pancreatitis	
0 Stage of subclinical chronic pancreatitis	
a Period without symptoms (determination by chance, e.g., autopsy)	
b Acute pancreatitis—single episode (possible onset of chronic pancreatitis)	
c Acute pancreatitis with severe complications^a^	
Symptomatic chronic pancreatitis	
I Stage without pancreatic insufficiency	
a (Recurrent) acute pancreatitis (no pain between episodes of acute pancreatitis)	
b Recurrent or chronic abdominal pain (including pain between episodes of acute pancreatitis)	
c I a/b with severe complications^a^	
II Stage of partial pancreatic insufficiency	
a Isolated exocrine (or endocrine) pancreatic insufficiency (without pain)	
b Isolated exocrine (or endocrine) pancreatic insufficiency (with pain)	
c II a/b with severe complications^a^	
III Stage of painful complete pancreatic insufficiency	
a Exocrine and endocrine insufficiency (with pain, e.g., requiring analgesic medication)	
b III a with severe complications^a^	
IV Stage of secondary painless disease (burnout)	
a Exocrine and endocrine insufficiency without pain and without severe complications^a^	
b Exocrine and endocrine insufficiency without pain and with severe complications^a^	

^a^Severe complications are defined as severe organ complications not included in the Cambridge classification. Reversible severe complications include development of ascites, bleeding, pseudoaneurysm, obstruction or stricture of the ductus choledochus, pancreatic fistula and duodenal stenosis. Irreversible severe complications are portal or splenic vein thrombosis with or without portal hypertension, and pancreatic cancer

#### Analyses

All analyses will be by intention-to-treat, meaning that all randomized patients are included in their initially assigned study arm. Baseline characteristics of the patients in the two study groups will be compared. For the analysis of the primary and secondary endpoints, we will use χ^2^ tests or Fisher’s exact test to analyze the different occurrence between the indomethacin group and the placebo group, with a final two-sided *P* value of less than 0.05 indicating statistical difference. That is, rectally administered indomethacin will be declared effective in the prevention of post-ESWL pancreatitis if *P* < 0.05. Results for the primary endpoint will be reported in terms of the absolute risk reduction (ARR), and relative risk reduction (RRR) with 95% CIs. Previous research has analyzed among the included potential risk factors including sex, presence of steatorrhea, pancreas divisum, frequent attacks of acute pancreatitis (at least one per year), diabetes, common bile duct (CBD) stenosis, alcohol consumption, multiple stones which were significantly different between patients with and without post-ESWL pancreatitis [9]. It has been stated that the incidence of PEP decreases as CP progresses, suggesting the clinical stage of CP to be an important factor in post-procedure pancreatitis [10]. Thus, an exploratory subgroup analysis by calculating relative risks was performed to assess whether the treatment effect differed in these prespecified factors mentioned above. Data will be analyzed using SPSS (version 22.0).

## Discussion

The RIPEP trial is designed to answer the question of whether rectally administered indomethacin can effectively decrease the incidence and severity of post-ESWL complications as well as associated adverse events, and we also want to prospectively investigate the risk factors for complications of P-ESWL, thus benefiting patients treated with P-ESWL.

With an incidence of more than 15% patients in high-risk patients, an effective prevention strategy of post-ERCP pancreatitis has been intensively investigated. The rectal administration of indomethacin and prophylactic pancreatic duct stenting has been supported by numerous high-quality studies [22]. Recently, a third potential method of intensive periprocedural fluid resuscitation has gained attention [23], showing that hydration affords protection against PEP. However, the included studies offered no clear guidance on the issues of the type of fluid, timing of hydration and a (re)hydration threshold. As the periprocedural fluid infusion strategy is same in the two arms, the study will offer no clear guidance on this issue, which needs further investigation.

Given the low cost, ease of administration and favorable side-effect profile, rectally administered indomethacin has aroused wide attention and the ESGE and ASGE guidelines have recommended it as a prophylactic method of PEP. A recently published meta-analysis of 4741 patients from 17 prospective trials showed that rectally administered diclofenac or indomethacin before, or closely after, ERCP were safe and effective in the prevention of PEP in all patients [24]. Based on pharmacokinetics, the peak plasma concentration of indomethacin and complete bioavailability can be reached 30 min after the rectal administration of indomethacin and the superior effect of its administration before ERCP was confirmed in high-risk patients [20, 25]. Thus, our study preferred the strategy of pre-procedural administration.

Pancreatic stones frequently develop during the natural course of CP and stones larger than 5 mm can be best fragmented by ESWL which is recommended as the first line therapy for CP [26]. Although reported as effective and safe, ESWL remains not complication-free. Like ERCP, among all the complications mentioned above, post-ESWL pancreatitis is the most common [7, 9]. Compared with all ESWL sessions, most complications occurred after first ESWL and pancreatitis predominates at rates reaching as high as 6.8%.

The RIPEP trial is a novel attempt to evaluate the effectiveness of rectally administered indomethacin in the era of NSAID prevention of PEP and an opportunity to fill in the gaps given that few studies have investigated the incidence and prophylaxis of post-ESWL complications while still emphasizing its safety and effectiveness. This is the first prospective, double-blinded, placebo-controlled trial to address related information about the complications of ESWL and its prevention. Despite the single study center, Changhai Hospital being the national center of CP management wherein the largest number of ESWL sessions in China are carried out, the result of the RIPEP trial will allow a broader assessment of the practice, augmenting the clinical generalizability of the prevention strategy.

Considering the high incidence rate of post-ESWL pancreatitis after the first ESWL procedure and its significant association with a higher risk for complications after the second ESWL session, we will particularly focus on the data after the first procedure.

In conclusion, a major focus of the RIPEP trial is to prospectively illustrate the effect of rectally administered indomethacin in reducing the development and severity of post-ESWL pancreatitis, and to identify the risk factors of developing complications and factors related to effective chemoprophylaxis, thus further benefiting patients treated with P-ESWL.

### Trial status

This randomized controlled trial began enrolling patients on 31 May 2016. As of April 2017, nearly 400 patients have been randomized and inclusion is on schedule.

## Additional file

Additional file 1: SPIRIT 2013 Checklist: recommended items to address in a clinical trial protocol and related documents*. (DOC 126 kb)

## Abbreviations

ARR: Absolute risk reduction
EPT: Endoscopic papillosphincterotomy
ERCP: Endoscopic retrograde cholangiopancreatography
MPD: Main pancreatic duct
NSAIDs: Nonsteroidal anti-inflammatory drugs
PEP: Post-ERCP pancreatitis
P-ESWL: Pancreatic extracorporeal shock wave lithotripsy
PPS: Prophylactic pancreatic stent
RIPEP: Rectally administered indomethacin to prevent post ESWL-pancreatitis
RRR: Relative risk reduction
TAE: Transient adverse event

## References

[1] Kim YH, Jang SI, Rhee K, Lee DK (2014). Endoscopic treatment of pancreatic calculi. Clin Endosc.

[2] Lawrence C, Siddiqi MF, Hamilton JN, Keane TE, Romagnuolo J, Hawes RH, Cotton PB (2010). Chronic calcific pancreatitis combination ERCP and extracorporeal shock wave lithotripsy for pancreatic duct stones. South Med J.

[3] Korpela T, Udd M, Tenca A, Lindstrom O, Halttunen J, Myrskysalo S, Mikkola A, Kylanpaa L (2016). Long-term results of combined ESWL and ERCP treatment of chronic calcific pancreatitis. Scand J Gastroenterol.

[4] Dumonceau JM, Delhaye M, Tringali A, Dominguez-Munoz JE, Poley JW, Arvanitaki M, Costamagna G, Costea F, Deviere J, Eisendrath P (2012). Endoscopic treatment of chronic pancreatitis: European Society of Gastrointestinal Endoscopy (ESGE) Clinical Guideline. Endoscopy.

[5] Tandan M, Reddy DN, Santosh D, Vinod K, Ramchandani M, Rajesh G, Rama K, Lakhtakia S, Banerjee R, Pratap N (2009). Extracorporeal shock wave lithotripsy and endotherapy for pancreatic calculi—a large single center experience. Indian J Gastroenterol.

[6] Hu L-H, Ye B, Yang Y-G, Ji J-T, Zou W-B, Du T-T, Hao J-F, Jiang Y-Y, Liao Z, Li Z-S (2016). Extracorporeal shock wave lithotripsy for Chinese patients with pancreatic stones. Pancreas.

[7] Li BR, Liao Z, Du TT, Ye B, Chen H, Ji JT, Zheng ZH, Hao JF, Ning SB, Wang D (2016). Extracorporeal shock wave lithotripsy is a safe and effective treatment for pancreatic stones coexisting with pancreatic pseudocysts. Gastrointest Endosc.

[8] Vaysse T, Boytchev I, Antoni G, Croix DS, Choury AD, Laurent V, Pelletier G, Buffet C, Bou-Farah R, Carbonnel F (2016). Efficacy and safety of extracorporeal shock wave lithotripsy for chronic pancreatitis. Scand J Gastroenterol.

[9] Li BR, Liao Z, Du TT, Ye B, Zou WB, Chen H, Ji JT, Zheng ZH, Hao JF, Jiang YY (2014). Risk factors for complications of pancreatic extracorporeal shock wave lithotripsy. Endoscopy.

[10] Zhao ZH, Hu LH, Ren HB, Zhao AJ, Qian YY, Sun XT, Su S, Zhu SG, Yu J, Zou WB, et al. Incidence and risk factors for post-ERCP pancreatitis in chronic pancreatitis. Gastrointest Endosc. 2017;86(3):519-24.e1. 10.1016/j.gie.2016.12.020 28062312

[11] Sun HL, Han B, Zhai HP, Cheng XH, Ma K (2014). Rectal NSAIDs for the prevention of post-ERCP pancreatitis: a meta-analysis of randomized controlled trials. Surgeon.

[12] Thiruvengadam NR, Forde KA, Ma GK, Ahmad N, Chandrasekhara V, Ginsberg GG, Ho IK, Jaffe D, Panganamamula KV, Kochman ML (2016). Rectal indomethacin reduces pancreatitis in high- and low-risk patients undergoing endoscopic retrograde cholangiopancreatography. Gastroenterology.

[13] Shi N, Deng L, Altaf K, Huang W, Xue P, Xia Q (2015). Rectal indomethacin for the prevention of post-ERCP pancreatitis: a meta-analysis of randomized controlled trials. Turk J Gastroenterol.

[14] Freeman ML (2016). Preventing post-ERCP pancreatitis: Update 2016. Curr Treat Options Gastroenterol.

[15] Fujisawa T, Kagawa K, Ochia K, Hisatom K, Kubota K, Sato H, Nakajima A, Matsuhashi N (2016). Prophylactic efficacy of 3- or 5-cm pancreatic stents for preventing post ERCP pancreatitis. J Clin Gastroenterol.

[16] Elmunzer BJ, Scheiman JM, Lehman GA, Chak A, Mosler P, Higgins PD, Hayward RA, Romagnuolo J, Elta GH, Sherman S (2012). A randomized trial of rectal indomethacin to prevent post-ERCP pancreatitis. N Engl J Med.

[17] Cote GA, Elmunzer BJ (2016). Nonsteroidal anti-inflammatory drugs for prevention of post-ERCP pancreatitis: sooner rather than later during ERCP?. Gastroenterology.

[18] Yokoe M, Takada T, Mayumi T, Yoshida M, Isaji S, Wada K, Itoi T, Sata N, Gabata T, Igarashi H (2015). Japanese guidelines for the management of acute pancreatitis: Japanese Guidelines 2015. J Hepatobiliary Pancreat Sci.

[19] Chandrasekhara V, Khashab MA, Muthusamy VR, Acosta RD, Agrawal D, Bruining DH, Eloubeidi MA, Fanelli RD, Faulx AL, Committee ASoP (2017). Adverse events associated with ERCP. Gastrointest Endosc.

[20] Luo H, Zhao L, Leung J, Zhang R, Liu Z, Wang X, Wang B, Nie Z, Lei T, Li X (2016). Routine pre-procedural rectal indomethacin versus selective post-procedural rectal indomethacin to prevent pancreatitis in patients undergoing endoscopic retrograde cholangiopancreatography: a multicentre, single-blinded, randomised controlled trial. Lancet.

[21] Schneider A, Lohr JM, Singer MV (2007). The M-ANNHEIM classification of chronic pancreatitis: introduction of a unifying classification system based on a review of previous classifications of the disease. J Gastroenterol.

[22] Wang AY, Strand DS, Shami VM (2016). Prevention of post-endoscopic retrograde cholangiopancreatography pancreatitis: medications and techniques. Clin Gastroenterol Hepatol.

[23] Smeets XJ, da Costa DW, Besselink MG, Bruno MJ, Fockens P, Mulder CJ, van der Hulst RW, Vleggaar FP, Timmer R, Drenth JP (2016). Systematic review: periprocedural hydration in the prevention of post-ERCP pancreatitis. Aliment Pharmacol Ther.

[24] Patai A, Solymosi N, Mohacsi L, Patai AV (2017). Indomethacin and diclofenac in the prevention of post-ERCP pancreatitis: a systematic review and meta-analysis of prospective controlled trials. Gastrointest Endosc.

[25] Wan J, Ren Y, Zhu Z, Xia L, Lu N (2017). How to select patients and timing for rectal indomethacin to prevent post-ERCP pancreatitis: a systematic review and meta-analysis. BMC Gastroenterol.

[26] Talukdar R, Reddy DN (2015). Pancreatic endotherapy for chronic pancreatitis. Gastrointest Endosc Clin N Am.

